# Microneedling for Anterior Neck Surgical Scars: A Systematic Review of Effectiveness and Evidence Gaps

**DOI:** 10.7759/cureus.84772

**Published:** 2025-05-25

**Authors:** Hassan Baig, Qaisar I Khan, Abdulaziz Al Failakawi, Mujahid Khan, James Lucocq

**Affiliations:** 1 Department of General Surgery, Queen Elizabeth University Hospital, Glasgow, GBR; 2 Department of Medical Education, University of Glasgow, Glasgow, GBR; 3 Department of General Surgery, Sabah Hospital, Kuwait, KWT; 4 Department of General Surgery, Victoria Hospital, Kirkcaldy, GBR

**Keywords:** aesthetic medicine, aesthetic outcome, hypertrophic scar, microneedling in scar management, surgical scar

## Abstract

Neck surgeries often leave anterior neck scars, causing cosmetic and psychological issues. These scars are more exposed to mechanical stress and the environment, potentially leading to hypertrophy, dyschromia, and scar widening. Traditionally, surgical scar interventions have been postponed until full scar maturation, usually between six and 12 months after surgery, but recent evidence indicates that early microneedling may improve collagen remodelling and scar quality. While microneedling has been studied for facial and abdominal scars, its effectiveness on anterior neck scars remains unclear. A systematic review was conducted following the Preferred Reporting Items for Systematic Reviews and Meta-Analyses (PRISMA) guidelines. Searches were performed in several databases and registers. Eligible studies included randomised controlled trials (RCTs), cohort studies, and observational studies assessing microneedling as a standalone intervention for surgical scars, using validated scar assessment tools such as the Patient and Observer Scar Assessment Scale (POSAS), the Vancouver Scar Scale (VSS), and the Global Aesthetic Improvement Scale (GAIS). Non-surgical scars, combination therapies, and animal studies were excluded. The database searches identified a total of 90 studies. After removing 13 duplicates, 77 studies underwent title and abstract screening, during which 68 were excluded due to irrelevance. The remaining nine articles were evaluated through full-text review; however, all nine articles were subsequently excluded for not meeting eligibility criteria. Ultimately, no studies specifically examining the effect of microneedling on anterior neck scars were eligible for inclusion. Although no direct studies on anterior neck scars exist, evidence from microneedling interventions on surgical scars in other body regions suggests potential benefits for anterior neck scars. Based on this indirect evidence, early intervention with three to six sessions at a depth of 1.0-1.5 mm may optimise scar outcomes. Further targeted research is required to confirm these findings and potentially establish microneedling as a standard management approach for scars following neck surgery.

## Introduction and background

Neck surgeries, such as thyroidectomies and parathyroidectomies, are frequently performed to treat both benign and malignant conditions, with post-operative scarring being an inevitable consequence [1,2]. The anterior neck is a visible and dynamic region, making surgical scars cosmetically and functionally significant. Despite advancements in surgical techniques, post-operative scarring remains a concern due to its visibility, exposure to environmental factors, and continuous movement.

Surgeons often attempt to place incisions within natural skin folds to minimise the visibility of scars; however, well-placed scars can still be noticeable, particularly in individuals with lighter or darker skin tones, where pigment changes are more evident [3]. Additionally, the thin skin of the anterior neck has lower collagen density and fewer sebaceous glands, making it prone to textural irregularities and prolonged erythema [4,5].

Unlike scars in less exposed anatomical areas, anterior neck scars are subjected to mechanical forces from daily head and neck movements, leading to scar widening, hypertrophy, and dyschromia [6,7]. These factors heighten patient dissatisfaction, particularly following elective surgeries, where cosmetic expectations are high [8,9].

Current advancements in neck surgeries emphasise scarless techniques, such as transoral or transaxillary approaches. However, these methods are not yet universally implemented due to practical constraints, reduced surgical efficiency, and economic considerations. Consequently, in situations where scarless surgery is not feasible, reducing the visibility and improving the quality of scars becomes essential to achieving optimal aesthetic outcomes and patient satisfaction.

Traditionally, scar management has been postponed for six to 12 months post-operatively to allow for complete scar maturation due to concerns that early interventions may disrupt healing. However, emerging evidence indicates that early interventions, such as microneedling, may enhance collagen remodelling and improve scar quality [10,11].

Microneedling, or percutaneous collagen induction therapy, is a minimally invasive technique, yielding promising results in enhancing scars by stimulating collagen and elastin production through controlled micro-injuries [12,13]. Studies on facial, chest, and abdominal surgical scars have demonstrated significant reductions in scar thickness, improved pigmentation, and enhanced texture, fostering optimism regarding their potential application in managing anterior neck scars [14,15]. Despite these encouraging findings, its role in addressing anterior neck scars specifically remains under-explored.

Given the cosmetic significance of anterior neck scars and the increasing interest in non-invasive techniques for scar enhancement, further research is crucial to determine optimal microneedling parameters, such as the timing of initiation, session frequency, duration, and needle depth [16]. This research will be pivotal for refining clinical practice and optimising post-operative scar management for patients undergoing neck surgery.

This systematic review aims primarily to provide a clear and focused evaluation of the effectiveness of microneedling for anterior neck surgical scars, in addition to assessing the optimal timing for initiating microneedling after surgery, comparing early intervention with traditional delayed treatment. Secondary outcomes include evaluating various treatment parameters, such as session frequency, duration, and needle depth, based on existing evidence from other surgical scars.

## Review

Methodology

Study Design

This systematic review followed the Preferred Reporting Items for Systematic Reviews and Meta-Analyses (PRISMA) guidelines to assess the effectiveness of microneedling for treating anterior neck surgical scars. The evaluation process upheld integrity and transparency.

Study Selection Criteria

The inclusion and exclusion criteria were generated using the Population, Intervention, Comparison, Outcomes, and Study design (PICOS) model, summarised in Table 1.

**Table 1:** Literature search inclusion and exclusion criteria PRP: platelet-rich plasma

	Inclusion	Exclusion
Patient	Adults > 16 years old, anterior neck surgical scars	Anyone < 16 years old, surgical scars other than the anterior neck
Intervention	Studies focusing on microneedling for anterior neck surgical scars	Studies evaluating microneedling in combination with other interventions, such as laser therapy or PRP. Any other treatment modalities
Control	Placebo or no other intervention	Studies evaluating microneedling in combination with other interventions, such as laser therapy or PRP. Any other treatment modalities
Outcome	Studies employing objective scar assessment tools, like the Patient and Observer Scar Assessment Scale (POSAS), Vancouver Scar Scale (VSS), or Global Aesthetic Improvement Scale (GAIS)	Studies not employing objective scar assessment
Study	Studies published in English within the past 25 years involving human subjects were eligible. Studies focussing on anterior neck surgical scars	Studies published in languages other than English, not involving human subjects. Studies focussing on areas other than anterior neck surgical scars.

This review included studies that specifically examined microneedling for surgical scars on the anterior neck only. Only studies employing objective scar assessment tools, like the Patient and Observer Scar Assessment Scale (POSAS), Vancouver Scar Scale (VSS), or Global Aesthetic Improvement Scale (GAIS), were eligible for selection. To maintain methodological rigour, we included randomised controlled trials (RCTs), cohort studies, observational studies, and case reports that evaluated microneedling as the sole treatment for anterior neck surgical scars. Only studies published in English within the past 25 years involving human subjects were eligible.

Studies were excluded if they focused on non-surgical scars, such as those resulting from acne, burns, or trauma, as these scars may respond differently to microneedling than surgical scars. Studies evaluating microneedling in combination with other interventions, such as laser therapy or platelet-rich plasma (PRP), were also excluded since these adjunctive treatments could confound the assessment of microneedling as an independent intervention. Moreover, this review did not include animal studies or conference abstracts lacking robust clinical evidence.

Search Strategy

A systematic search was conducted in the Cochrane Central Register of Controlled Trials, PubMed, Embase, Web of Science, and Google Scholar to identify relevant studies. The search was limited to articles published in English within the last 25 years. The reference lists of included studies were manually screened to identify additional relevant publications. Full search terms are detailed in Appendix 1.

Study Selection

**Figure 1:**
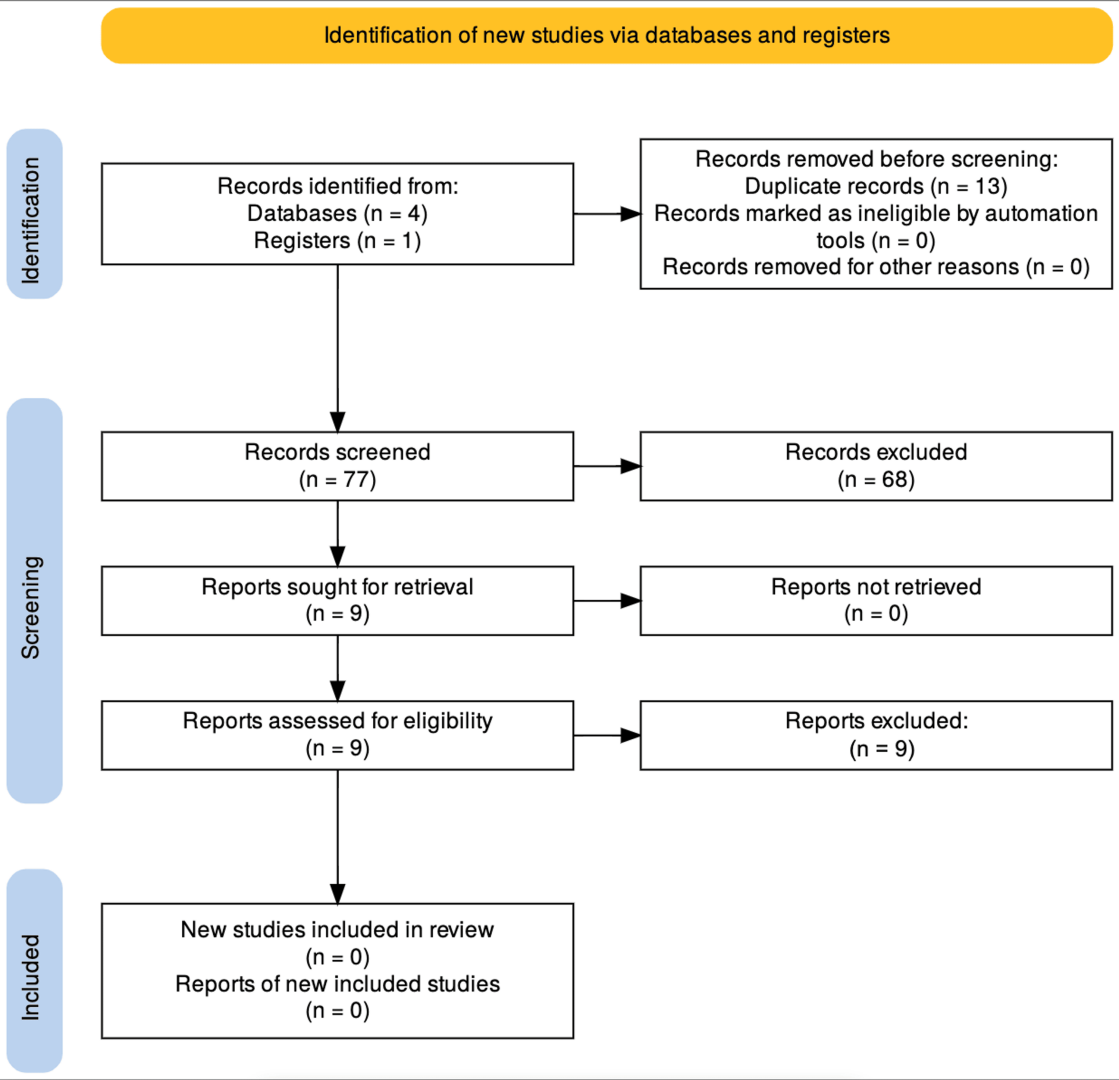
PRISMA flow diagram PRISMA: Preferred Reporting Items for Systematic Reviews and Meta-Analyses

A total of 90 records were identified through database and register searches (databases = 4, registers = 1). After removing 13 duplicate records, 77 were screened based on titles and abstracts. Of these, 68 were excluded due to irrelevance to the review focus. Nine full-text articles were assessed for eligibility, all of which were excluded for not specifically addressing anterior neck scar outcomes. Consequently, no studies met the inclusion criteria for this review (Figure 1).

Two independent reviewers then performed a thorough full-text assessment of the remaining nine articles using predefined eligibility criteria and the Scottish Intercollegiate Guidelines Network (SIGN) checklist for critical appraisal. Both reviewers agreed that none of these studies met the inclusion criteria, primarily due to the absence of research specifically evaluating microneedling for surgical scars on the anterior neck. Consequently, no studies were included in this systematic review (Appendix 2).

Results

No studies meeting the inclusion criteria were found, highlighting a significant gap in the literature regarding microneedling for anterior neck surgical scars. Given the established efficacy of microneedling in improving scars in other anatomical regions, there is an urgent need for further research to determine its effectiveness in anterior neck scars. This underscores the importance of our systematic review plan and its potential impact on scar management.

Discussion

Effectiveness of Microneedling

Studies evaluating microneedling for post-surgical scars have demonstrated significant reductions in standardised scar assessment scores. A study involving 25 patients with surgical scars found that three microneedling sessions, spaced four weeks apart, led to a 50% decrease in POSAS scores, from 23.7 ± 1.8 before treatment to 11.7 ± 1.0 at 16-week follow-up (p < 0.001) [17]. Similarly, an RCT assessing microneedling for post-abdominal surgical scars following deep inferior epigastric perforator (DIEP) flap-reconstruction found that treated scars exhibited a statistically significant improvement in POSAS scores at nine months post-treatment (median 17 vs. 21.4, p < 0.05) [18].

Although these studies did not focus on anterior neck surgical scars, they prove that microneedling can significantly enhance scar texture, pigmentation, and overall aesthetic appearance in post-surgical patients. Systematic reviews evaluating microneedling across various skin conditions, including atrophic acne scars, hypertrophic scars, keloids, and striae distensae, have also reported noteworthy improvements when microneedling is used alone or combined with adjunctive treatments. Importantly, these reviews highlight microneedling’s comparable effectiveness to other established modalities but with fewer adverse effects and shorter recovery periods, further supporting its potential application to visible and cosmetically sensitive areas like the anterior neck [19,20] Given that the neck is similarly exposed and holds high cosmetic importance, it is reasonable to hypothesise that microneedling would offer comparable benefits for anterior neck scars.

Given the similar cosmetic importance of anterior neck and facial scars, microneedling studies focusing on facial surgical scars are particularly relevant. One prospective study assessed microneedling outcomes across various scar types, including facial surgical scars, and found that all patients experienced at least a 50% improvement in scar appearance after an average of 2.5 treatments. Notably, over 80% of patients observed a 50% to 75% improvement, and 65% of patients demonstrated over 75% improvement in their scar scores (p < 0.001) [20]. These results indicate that microneedling is effective for surgical scars and especially advantageous for scars in prominent areas, such as the face and, by extension, the anterior neck.

Given that both facial and anterior neck scars share high visibility and patient concerns regarding aesthetic outcomes, these findings suggest that microneedling may be a valuable treatment for improving anterior neck scars following thyroidectomy, parathyroidectomy, and other cervical surgeries. However, due to the unique anatomical characteristics of the anterior neck, including thinner skin, fewer sebaceous glands, and increased movement, additional studies and targeted clinical trials focusing on optimal treatment parameters (needle depth, session frequency, and timing of initiation) for anterior neck scars are required to establish evidence-based clinical guidelines.

Optimal Timing for Initiating Microneedling Post-surgery

Traditionally, interventions to improve surgical scars have been delayed until the scar fully matures, typically between six and 12 months post-operatively. However, emerging evidence suggests that earlier initiation of microneedling may yield superior outcomes. One study evaluated the effects of microneedling on post-surgical scars, comparing early treatment (initiated at six to seven weeks post-operatively) with later treatment (commenced at 13 to 16 weeks post-operatively). The study utilised the POSAS to measure scar quality. Results indicated that the early treatment group experienced a significant improvement in POSAS scores, decreasing from 16.8 to 8.1, whereas the later treatment group showed a reduction from 26.1 to 14.2. The difference in improvement between the two groups was statistically significant (p < 0.04), suggesting that initiating microneedling earlier in the post-operative period may lead to better scar outcomes [21].

Needle Depth

The appropriate needle depth for microneedling varies depending on scar thickness, skin structure, and anatomical region. Studies on post-surgical facial and abdominal scars suggest that a depth of 1.5-2.0 mm effectively penetrates the dermis to stimulate collagen remodelling while minimising adverse effects such as prolonged erythema or epidermal damage (p < 0.05) [14]. Given that the anterior neck has thinner skin than the abdomen but shares similarities with the face, a needle depth of 1.0-1.5 mm may be optimal for microneedling in this area, allowing for adequate collagen stimulation without excessive tissue trauma.

Session Frequency

The frequency and total number of microneedling sessions significantly impact treatment efficacy. Studies assessing post-operative microneedling in surgical scars recommend a minimum of three to six sessions, spaced four to six weeks apart, to achieve statistically significant improvements in POSAS and VSS scores (p < 0.001) [22]. This interval allows for sufficient dermal remodelling between sessions while ensuring progressive scar pliability, pigmentation, and texture improvement. Given the high cosmetic importance of the anterior neck, a similar treatment schedule is likely appropriate for optimising scar remodelling in this region.

Overall, the findings highlight the potential role of microneedling as an effective intervention for surgical scars on the neck, with promising evidence from other surgical sites suggesting that early treatment initiation, appropriate needle depth, and optimal session frequency may significantly enhance scar remodelling and aesthetic outcomes. However, further research is required to establish definitive clinical guidelines specific to anterior neck scars.

## Conclusions

This review emphasises the potential effectiveness of microneedling as a minimally invasive option for treating surgical scars on the anterior neck. Although no direct studies have assessed its use in this area, evidence from other types of surgical scars indicates that microneedling can significantly enhance scar texture, pigmentation, and overall appearance, resulting in high patient satisfaction and minimal complications. Findings suggest that starting early microneedling treatment, approximately six weeks post-operatively, enhances scar remodelling compared to traditional delayed interventions. Moreover, treatment protocols consisting of three to six sessions, spaced four to six weeks apart, with needle depths adjusted to 1.0-1.5 mm, appear optimal for anterior neck scars. These parameters account for the uniquely thin and delicate nature of the anterior neck skin, thus maximising therapeutic efficacy. Clinicians should consider integrating microneedling into standard neck surgery scar management protocols to enhance aesthetic outcomes. Further research is needed to establish long-term effects beyond 12 months and to develop specific guidelines for anterior neck scars. Future studies should focus on RCTs to confirm ideal treatment timing, frequency, and needle depth for this anatomical region.

## Appendices

Appendix 1

**Table 2:** Search terms summary

Database	Search strategy
PubMed	("Cicatrix"[MeSH] OR "scar"[tiab] OR "surgical scar"[tiab] OR "postoperative scar"[tiab] OR "wound healing"[tiab]) AND ("Microneedling"[MeSH] OR "microneedling"[tiab] OR "collagen induction therapy"[tiab] OR "skin needling"[tiab] OR "percutaneous collagen induction"[tiab]) AND ("Patient Satisfaction"[MeSH] OR "patient satisfaction"[tiab] OR "treatment outcome"[tiab] OR "aesthetic outcome"[tiab] OR "aesthetic improvement"[tiab] OR "cosmetic result"[tiab]) AND ("Wound Healing"[MeSH] OR "skin regeneration"[tiab] OR "scar remodeling"[tiab] OR "skin texture"[tiab] OR "skin repair"[tiab])
Embase	'surgical scar'/exp OR 'postoperative complication'/exp OR 'cicatrix'/exp OR (surgical scar* OR postoperative scar* OR neck scar* OR cicatrix* OR wound healing):ti,ab AND ('neck surgery'/exp OR 'anterior neck':ti,ab OR 'cervical region':ti,ab OR 'neck incision':ti,ab) AND 'microneedling'/exp OR (microneedling OR "collagen induction therapy" OR "skin needling" OR "percutaneous collagen induction"):ti,ab AND 'patient satisfaction'/exp OR 'treatment outcome'/exp OR 'aesthetic result'/exp OR (patient satisfaction OR treatment outcome* OR aesthetic outcome* OR cosmetic result*):ti,ab AND (POSAS OR "Patient and Observer Scar Assessment Scale" OR "Vancouver Scar Scale" OR VSS OR "Global Aesthetic Improvement Scale" OR GAIS):ti,ab
Web of Science	TS=("surgical scar*" OR "postoperative scar*" OR "wound healing" OR "neck scar*") AND TS=("anterior neck" OR "neck incision" OR "neck surgery" OR "cervical region") AND TS=("microneedling" OR "collagen induction therapy" OR "skin needling" OR "percutaneous collagen induction") AND TS=("patient satisfaction" OR "aesthetic outcome*" OR "cosmetic result*" OR "treatment outcome*") AND TS=("POSAS" OR "Vancouver Scar Scale" OR VSS OR GAIS)
Cochrane Central Register of Controlled Trials (CENTRAL)	("surgical scar" OR "postoperative scar" OR "neck scar" OR cicatrix OR "wound healing") AND ("anterior neck" OR "neck incision" OR "neck surgery" OR "cervical region") AND (microneedling OR "collagen induction therapy" OR "skin needling" OR "percutaneous collagen induction") AND ("patient satisfaction" OR "treatment outcome" OR "aesthetic outcome" OR "cosmetic result") AND (POSAS OR "Patient and Observer Scar Assessment Scale" OR "Vancouver Scar Scale" OR VSS OR "Global Aesthetic Improvement Scale" OR GAIS)
Google Scholar	("surgical scar" OR "postoperative scar" OR "neck scar" OR "scar after surgery" OR "wound healing") AND ("anterior neck" OR "neck incision" OR "neck surgery" OR "cervical region") AND (microneedling OR "collagen induction therapy" OR "skin needling") AND ("aesthetic outcome" OR "cosmetic appearance" OR "patient satisfaction") AND ("POSAS" OR "Vancouver Scar Scale" OR GAIS OR "scar appearance")

Appendix 2 

**Table 3:** Full-text studies excluded from review and rationale

Title	Authors	Year	Reason for exclusion
Microneedling: A Review and Practical Guide	Alster et al.	2018	Did not explicitly evaluate anterior cervical scar outcomes
Microneedling for the Treatment of Scars: An Update for Clinicians	Juhasz et al.	2020	Did not explicitly evaluate anterior cervical scar outcomes
Microneedling of Scars: A Large Prospective Study With Long-Term Follow-Up	Alster et al.	2020	Did not explicitly evaluate anterior cervical scar outcomes
Microneedling Outcomes in Early Postsurgical Scars	Claytor et al.	2022	Did not explicitly evaluate anterior cervical scar outcomes
Evaluation of Microneedling Therapy in Management of Facial Scars	Vijaya Lakshmi et al.	2023	Did not explicitly evaluate anterior cervical scar outcomes
Clinical Evaluation of Microneedling Therapy in the Management of Facial Scars: A Prospective Randomized Study	Bandral et al.	2023	Did not explicitly evaluate anterior cervical scar outcomes
Percutaneous Collagen Induction Therapy: An Alternative Treatment for Scars, Wrinkles, and Skin Laxity	Aust et al.	2023	Did not explicitly evaluate anterior cervical scar outcomes
Microneedling in Abdominal Scarring After DIEP-Flap Breast Reconstruction to Improve Scar Quality: A Randomized Controlled Split Scar Trial	Everaars et al.	2024	Did not explicitly evaluate anterior cervical scar outcomes
Microneedling Monotherapy Versus Microneedling With Autologous Platelet-Rich Plasma for the Treatment of Stretch Marks (Striae Distensae) and Post-surgical Scars	Kaur et al.	2024	Did not explicitly evaluate anterior cervical scar outcomes
